# Anion Exchange HPLC Isolation of High-Density Lipoprotein (HDL) and On-Line Estimation of Proinflammatory HDL

**DOI:** 10.1371/journal.pone.0091089

**Published:** 2014-03-07

**Authors:** Xiang Ji, Hao Xu, Hao Zhang, Cheryl A. Hillery, Hai-qing Gao, Kirkwood A. Pritchard

**Affiliations:** 1 Department of Geriatrics, Qilu Hospital, Shandong University, Key Laboratory of Cardiovascular Proteomics of Shandong Province, Jinan, Shandong, China; 2 Department of Surgery, Division of Pediatric Surgery, Medical College of Wisconsin, Milwaukee, Wisconsin, United States of America; 3 Department of Pediatrics, Division of Hematology, Medical College of Wisconsin, Milwaukee, Wisconsin, United States of America; 4 Blood Research Institute, Milwaukee, Wisconsin, United States of America; 5 Children’s Research Institute, Milwaukee, Wisconsin, United States of America; 6 Medical College of Wisconsin, Milwaukee, Wisconsin, United States of America; University of South Florida College of Medicine, United States of America

## Abstract

Proinflammatory high-density lipoprotein (p-HDL) is a biomarker of cardiovascular disease. Sickle cell disease (SCD) is characterized by chronic states of oxidative stress that many consider to play a role in forming p-HDL. To measure p-HDL, apolipoprotein (apo) B containing lipoproteins are precipitated. Supernatant HDL is incubated with an oxidant/LDL or an oxidant alone and rates of HDL oxidation monitored with dichlorofluorescein (DCFH). Although apoB precipitation is convenient for isolating HDL, the resulting supernatant matrix likely influences HDL oxidation. To determine effects of supernatants on p-HDL measurements we purified HDL from plasma from SCD subjects by anion exchange (AE) chromatography, determined its rate of oxidation relative to supernatant HDL. SCD decreased total cholesterol but not triglycerides or HDL and increased cell-free (cf) hemoglobin (Hb) and xanthine oxidase (XO). HDL isolated by AE-HPLC had lower p-HDL levels than HDL in supernatants after apoB precipitation. XO+xanthine (X) and cf Hb accelerated purified HDL oxidation. Although the plate and AE-HPLC assays both showed p-HDL directly correlated with cf-Hb in SCD plasma, the plate assay yielded p-HDL data that was influenced more by cf-Hb than AE-HPLC generated p-HDL data. The AE-HPLC p-HDL assay reduces the influence of the supernatants and shows that SCD increases p-HDL.

## Introduction

High-density lipoprotein (HDL) serves many functions that play important roles in preserving vascular health. HDL pickups cholesterol from the periphery and carries it to the liver for excretion via the reverse cholesterol transport pathway, [1] inhibits LDL oxidation, [2] and even stimulates vascular endothelial cell generation of nitric oxide. [3], [4] However, in disease, such as occurs in patients with coronary artery disease, [5], [6] diabetes, [7] influenza, [8] lupus, [9] scleroderma [10] and sickle cell disease [11] chronic states of oxidative stress increase HDL oxidative modification and impair function. When HDL fails to properly perform its many duties or induces atherogenic responses it is considered proinflammatory. [12].

Estimates of proinflammatory HDL (p-HDL) employ bioassays that quantify HDL’s ability to inhibit LDL oxidation, [13] take up and release cholesterol [4] or inhibit oxidized LDL-induced monocyte chemotaxis. [8] One of the consistent biochemical features of p-HDL is an increase in the number of seeding molecules of oxidized lipids that have been shown to correlate with HDL bioassay data. Realizing the need for a more convenient means of quantifying p-HDL Navab et al. [14] developed a cell-free assay that showed that p-HDL was increased in cardiovascular patients at risk of atherosclerosis when compared with p-HDL in controls. Protocols for quantifying p-HDL often call for precipitation of apo B containing lipoproteins, addition of a lipid hydroperoxide and oxidizable phospholipids or LDL, that serve as an oxidant challenge and dichlorofluorescein (DCFH), an oxidation sensitive fluorescent probe that aids in determining rates of HDL oxidation. Using a modification of the Navab cell-free assay [14] where Cu^2+^ was used as an oxidant, we showed that p-HDL was increased in tight skin mice, an established murine model of scleroderma and in humans with scleroderma. [10] While precipitating apo B lipoproteins is a convenient technique for rapidly isolating HDL, we reasoned that in some patient populations apo B precipitation would yield complex supernatant matrices that might influence rates of HDL oxidation.

Sickle cell disease is a major focus of our research program. Patients with SCD suffer from repeated bouts of ischemia/reperfusion injury resulting in intravascular hemolysis and release of xanthine oxidase (XO) from injured tissues.[15]–[17] As XO generates superoxide anion which dismutates into hydrogen peroxide and cell-free hemoglobin (cf Hb) can degrade hydrogen peroxide into hydroxyl radicals, [18] it is possible that plasma supernatants from SCD patients will contain these pro-oxidant components and therefore artificially increase p-HDL. To test this possibility we isolated HDL by AE-HPLC, oxidized it and mixed it with DCFH using a dual post-column reactor (PCR) and then measured levels of HDL DCF fluorescence. Our findings show that after apoB precipitation the supernatants from SCD patients increase p-HDL when determined using a plate assay format and that cf Hb and XO are capable of teaming up to artificially increase p-HDL.

## Materials and Methods

### Ethics Statement

Approval for the studies involving samples from human subjects was obtained from the *Institutional Review Board* of Children’s Hospital of Wisconsin and the Medical College of Wisconsin (MCW). Signed and written informed consent forms were obtained from control subjects and patients and/or guardians of children and all participants in the study. The study and protocols for obtaining written consent forms for the participants were approved by the *Institutional Review Board* of Children’s Hospital of Wisconsin. The murine plasma studies were approved by the *Institutional Animal Care and Use Committee* of MCW. This study was carried out in strict accordance with the recommendations in the Guide for the Care and Use of Laboratory Animals of the National Institutes of Health. All surgical protocols used to obtain the plasma were performed under complete ketamine:xylazine anesthesia and all efforts were made to minimize pain and suffering.

### Human Subjects

All patients in this study had either Hb SS disease or Hb S-βo thalassaemia as documented by quantitative Hb electrophoresis or high-performance liquid chromatography (HPLC). Patients attended the Wisconsin Sickle Cell Disease Comprehensive Center in Milwaukee, WI as we have described previously. [19].

### Mouse

Plasma was pooled from C57BL/6J mice (controls) that were euthanized as part of on-going studies examining vascular reactivity for another project.

### Plasma Lipids

Total cholesterol, triglycerides and HDL cholesterol (HDL_c_) were quantified using reagents from Wako (Richmond, VA, USA). Plasma apolipoprotein A-I levels were quantified using an ELISA kit from MABTECH, Inc. (Cincinnati, OH, USA).

### Proinflammatory HDL

A modified method of the Navab the cell-free assay [14] was used to quantify p-HDL. The modified protocol called for apo B containing lipoproteins to be precipitated from plasma with dextran-sulfate/MgCl_2_ (DS) precipitating reagent. HDL oxidation was initiated with Cu^2+^ and rates of HDL oxidation quantified with DCFH as previously described. [10] Details of the standard plate protocol were as follows: Two mg of 2′,7′-dichlorofluorescin diacetate (Calbiochem, Inc., San Diego, CA) was dissolved in 1 mL of methanol, incubated for 30 min at room temperature and then diluted 10X with deionized water to a final concentration of 0.2 mg/mL. An aliquot of the plasma supernatant containing 1 µg of HDL cholesterol was incubated with CuCl_2_ (5 µM, final concentration) for 1 h in a blackwell 384-well microtiter plate to initiate oxidation. Next, 10 µL of DCFH (0.2 mg/mL) was added to the HDL-Cu^2+^ mixture in a total volume of 50 µL. DCF fluorescence (Ex 488 nm/Em 530 nm) was measured at 30 min intervals over the next 2 h at room temperature. Relative rates of DCF fluorescence were calculated. Data were presented as mean ± SD.

### AE-HPLC Isolation of HDL and Estimates of p-HDL

Plasma lipoproteins were separated using a modified AE chromatography originally described by Hirowatari et al. [20] The HPLC workstation consisted of an Agilent 1100 with an autoinjector (G1313A), diode array detector (G1315A) and fluorescent detector (G1321A). Separation was accomplished using an analytical 4.6 mm ID×3.5 cm TSK column fitted with a 4.6 mm ID×5 µm TSK pre-guard column from Tosoh (King of Prussia, PA) and disposable Opti-guards (Optimized Technologies 10-02-00030, Oregon City, OR) which were changed every 30 injections. Buffer A was HPLC-grade water containing Tris-HCl (pH = 7.5, 50 mM) and Na_2_EDTA (1 mM). Buffer B was Buffer A containing NaClO_4_ (500 mM). Both buffers were purged with argon for 5 min prior to use. To isolate HDL, ionic strength of the elution buffer was altered by changing % B Buffer as follows: Step 1) increase B Buffer from 0 to 7% linearly over 5 min; step 2) hold % B Buffer constant at 7% for 15 min; step 3) increase % B Buffer to 18%, step 4) hold % B Buffer constant at 18% for 15 min to elute HDL, step 5) increase % B Buffer to 100% over 8 min; step 6) hold % B Buffer constant for 10 min to wash the column and then step 7) reduce % B Buffer to 0% and hold constant for 10 min to regenerate the column. The flow rate of the mixed buffers running through the analytical columns was held constant at 0.5 mL/min. NaClO_4_ was used to adjust the ionic strength of the eluent because NaClO_4_ is a chaotropic salt, that increases the water solubility of hydrophobic particles [21] similar to lipoproteins. Using NaClO4 for AE buffers improves separation of the lipoproteins by reducing nonspecific interactions between the lipoproteins and the surface of the DEAE gel as was shown previously by Hirowatari et al. [20] To visualize lipoprotein separation DiI (10 µg/mL, final concentration, Sigma-Aldrich, cat#42364) was added and mixed with plasma samples. Whenever DCF was used to measure p-HDL levels, DiI was not added to the plasma samples because the fluorescent spectra of DiI conflicted with the fluorescent spectra of oxidized DCF. The AE-HPLC-PCR was allowed to equilibrate in 0% B Buffer for at least 30 min prior to injecting the next sample. Post-column reactions to estimate p-HDL took place in a Sensivate PCR II dual pump post-column reactor (Hitachi High Technology America, Schomberg, IL). The first PCR reactor (50°C) mixed column effluent with Cu^2+^ (100 µM) infused at the rate of 0.05 mL/min such that the final concentration of Cu^2+^ in the reactor line would equal 10 µM. The second PCR reactor (50°C) was infused with DCFH in methanol (0.05 mL/min) such that its final in-line concentration would equal 0.1 mg/mL. The DCFH solution was kept in the dark with storage bottles and reagent lines being wrapped in aluminum foil. Plasma (30 µL) was injected and DCF fluorescence intensity (based on area) in the HDL peaks was divided by plasma apoA-I concentrations. Results were expressed as mean ± SD (RFU/apoA-I (µg/mL)).

### Purification of HDL

Purified human HDL was isolated from pooled human plasma by sequential density ultracentrifugation (d = 1.063–1.21 g/mL) as previously described. [22].

### Plasma Cell-free Hemoglobin

cf Hb was quantified using reagents from Catachem (Oxford, CT, USA) as previously described. [23].

### Plasma Xanthine Oxidase

Plasma XO was quantified by immunoblot analysis as previously described. [24], [25].

### Effects of Hemoglobin and Xanthine Oxidase on HDL Oxidation

HDL (0.01 mg/mL cholesterol, final) was incubated with XO (14.3 µU/mL, final, Sigma-Aldrich, X4500, St. Louis, MO), xanthine (0.02 µM, final, Sigma-Aldrich, X7379, St. Louis, MO), and Hb (0, 4 and 16 mg/dL, final, Sigma-Aldrich, X7375) at room temperature for 100 min, during which A_234 nm_ was recorded by Agilent 8453 UV-Vis spectrophotometer (Agilent Technologies, Santa Clara, CA). The initial rate of HDL conjugated diene formation/oxidation was expressed as the slope of linear regression of A_234 nm_ over the first 40 min.

### Immunoblot Analysis

Sample fractions were collected from the HPLC effluent based on peak position and concentrated using Centrifugal Filter Units (Regenerated Cellulose 3000 MWCO, Amicon Ultra, Billerica, MA). Concentrates were examined by immunoblotting for apoA-I (sc30089), Hb (sc21005, Santa Cruz Biotechnology Inc., Paso Robles, CA), XO (MS-474-P, NeoMarkers, Fremont, CA) and albumin (ab106585, Cambridge, MA).

### Statistical Analysis

Data are presented as mean ± SD unless otherwise indicated. Data from two populations were analyzed by the student’s t-test while data from more than two populations were analyzed by ANOVA with post-hoc analysis performed using Bonferroni’s method for correcting for multiple comparisons to estimate significance between different populations.

## Results

**Figure 1:**
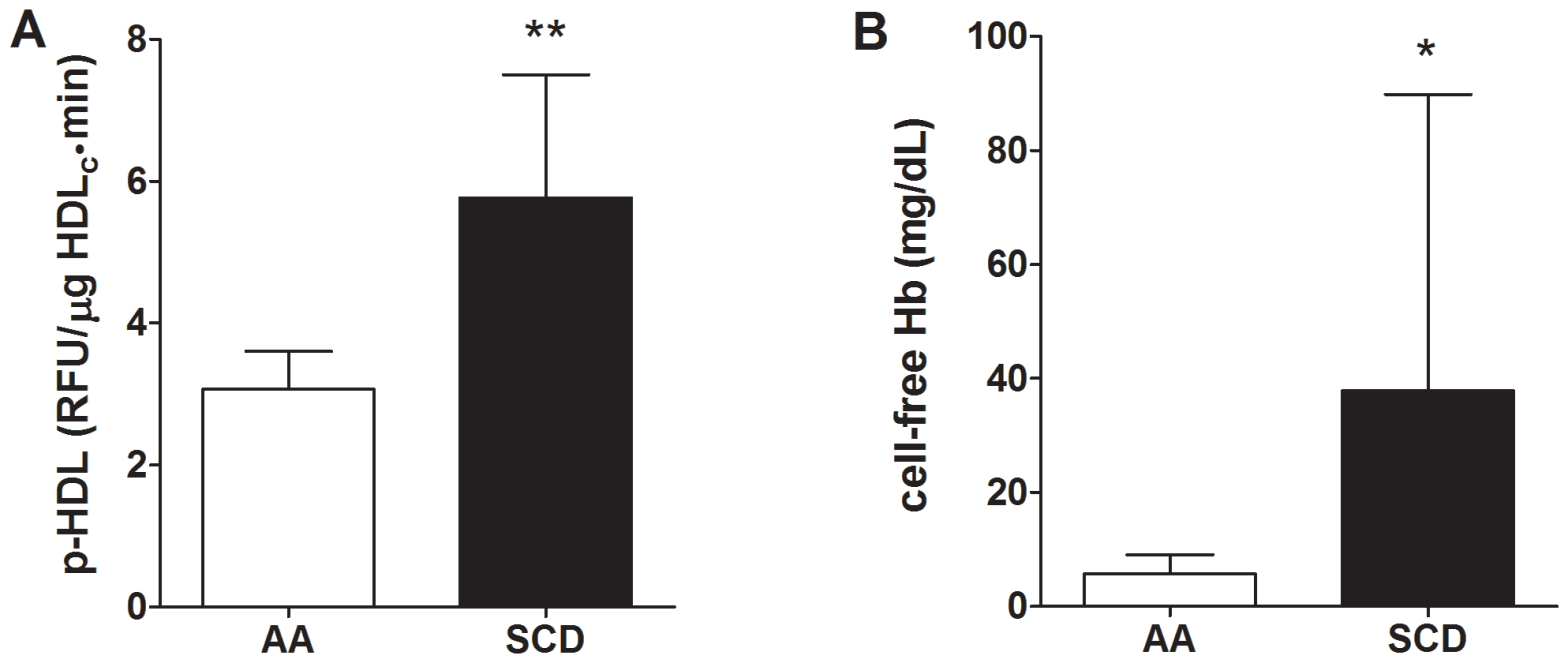
Effects of SCD on p-HDL and cf Hb. (A) Proinflammatory HDL (p-HDL) in SCD patients was increased compared with controls (** = p<0.01, n = 6). (B) Cell-free (cf) Hb was increased in SCD patients compared with controls (* = p<0.05, n = 6).

**Figure 2:**
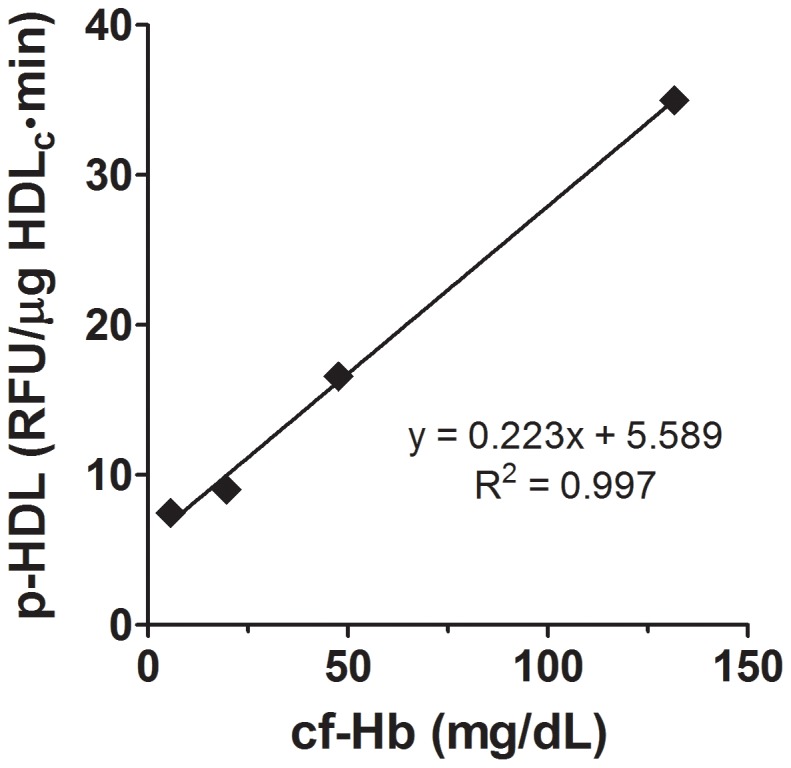
Effects of cf Hb on p-HDL. P-HDL levels were determined by the plate assay. Pooled mouse plasma was analyzed for cf Hb (5.6 mg/dL) and p-HDL (7.46 RFU/min). Increasing concentrations of human Hb were added to the mouse plasma, apoB lipoproteins precipitated and supernatant p-HDL levels determined immediately after apo B precipitation. Plotted data were mean of duplicates.

SCD decreased total plasma cholesterol compared to non-sickle controls (p<0.01) but had no effect on plasma triglycerides or HDL_c_ (**Table 1**). SCD however, increased p-HDL as measured by the plate assay compared with controls (**Figure 1A**). Plasma cf Hb was increased in SCD human samples compared to controls (**Figure 1B**). To determine if plasma cf Hb interfered with p-HDL measurements by the plate assay, control mouse plasma was first analyzed for baseline levels of cf Hb (5.6 mg/dL); HDL_c_ (35.1 mg/dL) and p-HDL (7.46 RFU/min). Human cf Hb was added to the mouse plasma, mixed, apoB lipoproteins precipitated and p-HDL levels determined immediately. Hb increased p-HDL in direct relation to final cf Hb concentrations (**Figure 2**). These data suggest that any increase in cf Hb that results from intravascular hemolysis in SCD patients during vaso-occlusive crisis or from red cell lysis during phlebotomy may artificially increase p-HDL when using the plate assay.

**Table 1:** Plasma lipid analysis: Cholesterol, Triglycerides and HDL cholesterol (HDL_c_) for Control subjects and SCD patients (n = 6).

Test Group	Cholesterol (mg/dL)	Triglycerides (mg/dL)	HDL_c_ (mg/dL)
Control (n = 6)	151.2±22.5	89.0±74.6	37.5±9.6
SCD (n = 6)	119.8±18.9	60.1±27.9	33.0±7.7
P value	0.01	0.20	0.20

**Figure 3:**
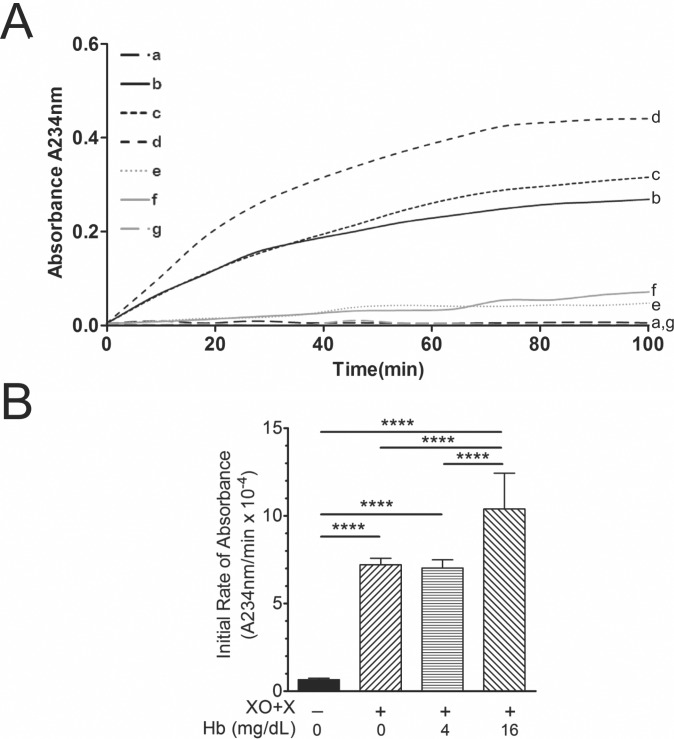
Effects of XO, X, cf Hb alone and in combination on HDL conjugated diene formation. (A) XO, X, cf Hb alone and in combination were added to purified human HDL and initial rates of absorbance (A_234 nm_) recorded over time. Absorbance at 234 nm was recorded and initial rates of absorbance calculated per test group. (A) Line graphs showing changes in A_234 nm_ with respect to time for the following test groups: a) HDL alone; b) HDL+XO+X; c) HDL+Hb (4 mg/dL) +XO+X; d) HDL+Hb (16 mg/dL) +XO+X; e) HDL+X; f) HDL+XO; and, g) XO+X. (B) The bar chart showed that XO/X increases A_234 nm_ at faster rates than HDL alone and that adding cf Hb increased initial rates of HDL oxidation greater than XO/X alone when cf Hb equals 16 mg/dL but, not 4 mg/dL. (**** = p<0.001, n = 5–12).

SCD increases oxidative stress by increasing the release of XO from injured tissues. [26] Adding XO+X to purified HDL increases initial rates of HDL conjugated diene formation (**Figure 3A and 3B**). Adding XO or X alone has little, if any effect, on the initial rates of HDL conjugated diene formation (**Figure 3A**). Adding XO+X to solutions of HDL in the presence of cf Hb further increases the initial rates of HDL conjugated diene formation when cf Hb equals 16 mg/dL but not 4 mg/dL (**Figure 3A and 3B**).

**Figure 4:**
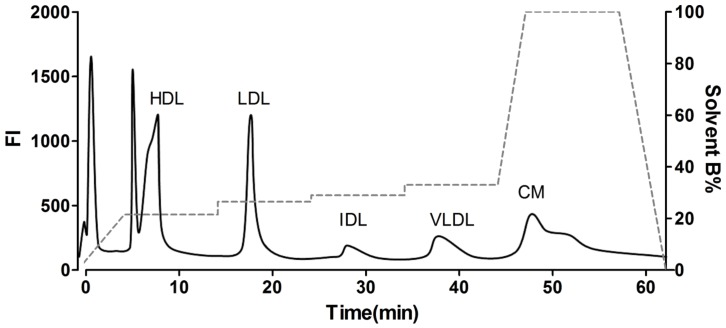
AE-HPLC Protocol for Separating Lipoproteins. DiI (10 µg/mL, final concentration) was added to plasma and lipoproteins separated by AE-HPLC using the protocol published by Hirowatari et al.^20^ This DiI chromatogram shows that all 5 classes of lipoproteins are separated as reported.^20^ However, the chromatogram also shows that DiI associates with two other components, at the very front of the chromatogram and in a peak on the shoulder of the HDL peak.

AE-HPLC has been used to separate plasma lipoproteins for over 10 years. [20] Lipophilic fluorescent dyes, such as DiI have been used to label lipoproteins even longer. [27] To monitor lipoprotein separation we treated plasma samples with DiI prior to AE-HPLC analysis. A representative AE-HPLC DiI chromatogram using the Hirowatari et al. [20] protocol can be seen in **Figure 4**. Although treating plasma samples with DiI made it easy to monitor elution of the 5 different lipoprotein classes, this chromatogram shows that factors or elements other than lipoproteins also appear to bind DiI.

**Figure 5:**
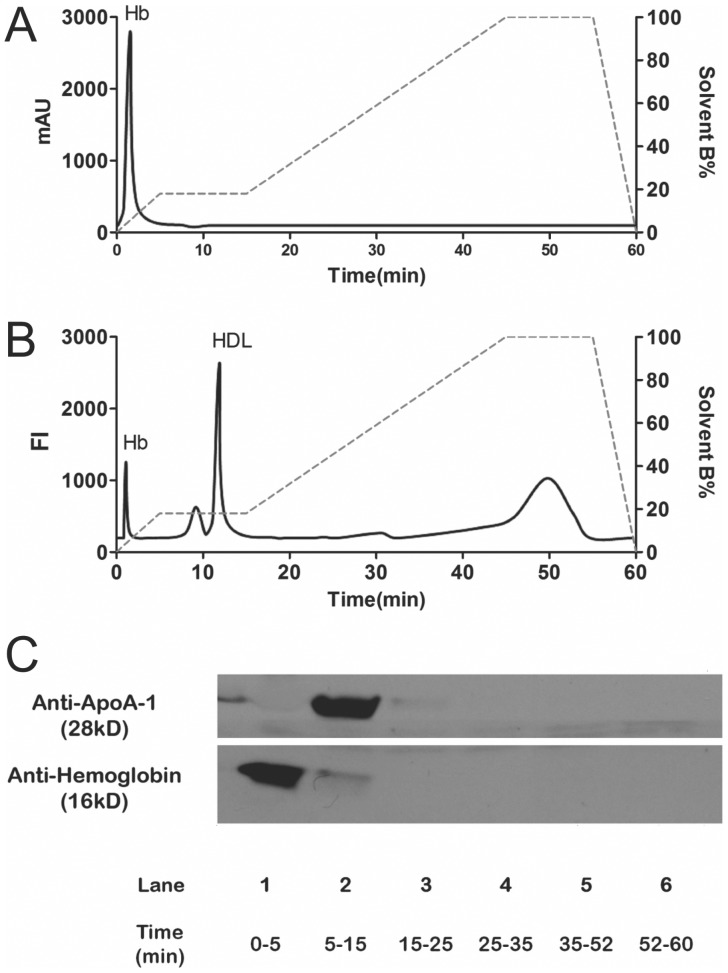
AE-HPLC separation of cf Hb and HDL from plasma. AE-HPLC of Hb alone revealed Hb elutes at 0–5 min. (A) Cf Hb (100 mg/dL) 30 µl was injected without DiI, which was measured by UV absorbance (A_230 nm_). (B) Plasma treated with DiI (10 µg/mL) and then 30 µL was analyzed by fluorescent AE-HPLC (Ex 530 nm/Em 577 nm). (C) Plasma (30 µL) was injected, fractions collected, pooled, concentrated and examined by immunoblot analysis for apoA-I and Hb.

To determine if the first unknown fluorescent peak in the DiI chromatogram (**Figure 4** **)** contained Hb, we injected purified cf Hb (100 mg/dL). The UV absorbance chromatogram (**Figure 5A**) shows a single peak that elutes before 5 min, which is essentially at the same time point as the first unknown peak in **Figure 4**. The retention times for cf Hb and DiI-treated cf Hb were confirmed in subsequent studies (see **Figure S1**). To confirm that the elution protocol separates cf Hb from HDL, DiI was added to plasma and the mixture analyzed by AE-HPLC. The DiI chromatogram in **Figure 5B** shows the fluorescence intensity of the eluting peaks while the immunoblots in **Figure 5C** demonstrate that Hb elutes in the 0–5 min pooled fraction and that HDL, measured as apoA-I, elutes in the 5–15 min pooled fraction.

**Figure 6:**
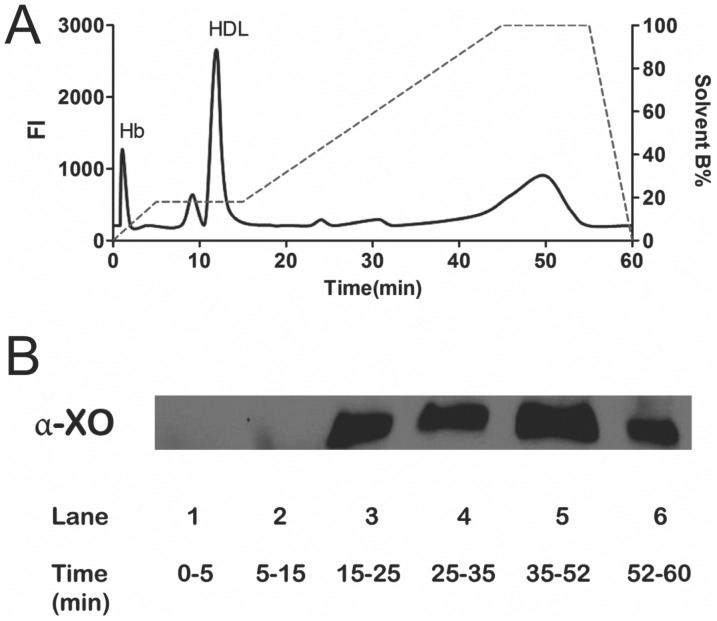
Separation of Xanthine Oxidase (XO) from HDL. DiI-treated plasma was injected (30 µL) into the AE-HPLC. Fractions were collected, concentrated and examined by immunoblot for XO. The chromatogram in A and immunoblot in B showed that the modified AE-HPLC protocol eluted HDL prior to XO.

In the presence of xanthine (X), xanthine oxidase (XO) can generate high levels of superoxide anion and hydrogen peroxide to increase oxidative stress. To determine where XO elutes in the AE-HPLC profile we injected DiI plasma, collected and pooled fractions and ran immunoblots for XO. The fluorescent intensity chromatogram in **Figure 6A** shows the typical elution profile for Hb and HDL. The XO immunoblot in **Figure 6B** shows that XO elutes starting at 15–25 minutes and longer which is well after the time HDL is known to elute. Exactly why XO is observed in multiple pooled fractions with increasing ionic strength is unclear at this time but these data suggest that XO has high affinity to AE column that is not easily disrupted.

**Figure 7:**
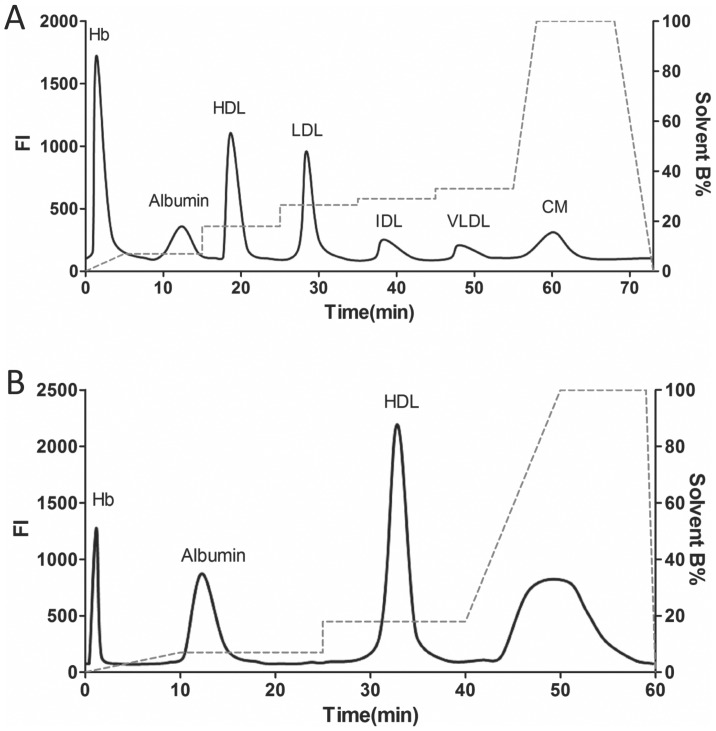
Standardized Lipoprotein and HDL Elution Profiles. (A) DiI chromatogram for AE-HPLC separation of Hb, Albumin and the 5 lipoprotein (HDL, LDL, IDL, VLDL, CM) fractions. (B) DiI chromatogram for AE-HPLC separation of Hb, Albumin and HDL.

Albumin is also known to bind lipophilic dyes, [28] fatty acids and lipid hydroperoxides [29] as well as heme [29] which can interfere with AE-HPLC assay for p-HDL at multiple levels. When albumin binds DiI and DCF, it gives the appearance of a “new” false peak in the lipoprotein chromatogram. By carrying lipid hydroperoxides or heme albumin has the potential to increase oxidation of HDL by mechanisms that have nothing to do with the number of seeding molecules of lipid hydroperoxides [30] in HDL that are considered responsible for enhancing the proinflammatory character of HDL and therefore its p-HDL value. Assuming that the second unknown peak might be albumin, we systematically modified the protocol to enhance separation (see **Figure S2**) and then performed immunoblots on the pooled fractions from a series of AE-HPLC runs of plasma to determine which % B buffer concentration was the best for separating albumin from HDL. Our studies showed that ramping % B buffer up to 7 and holding it steady at 7% for 15 min was the best for separating albumin from HDL (**Figure 7A**). Using this protocol, which is modified from the one reported by Hirowatari et al. [20] we were able to successfully separate Hb, albumin as well as all 5 classes of lipoproteins (**Figure 7A**). As our studies here focused on using AE-HPLC to isolate HDL, we next shortened the AE-HPLC protocol to ensure HDL could be isolated from Hb, albumin and the other lipoproteins (**Figure 7B**). The importance of using this approach is demonstrated by the fact that HDL purified by AE-HPLC is essentially free from XO and Hb which is not the case with HDL isolated by DS (**Figure S3**).

**Figure 8:**
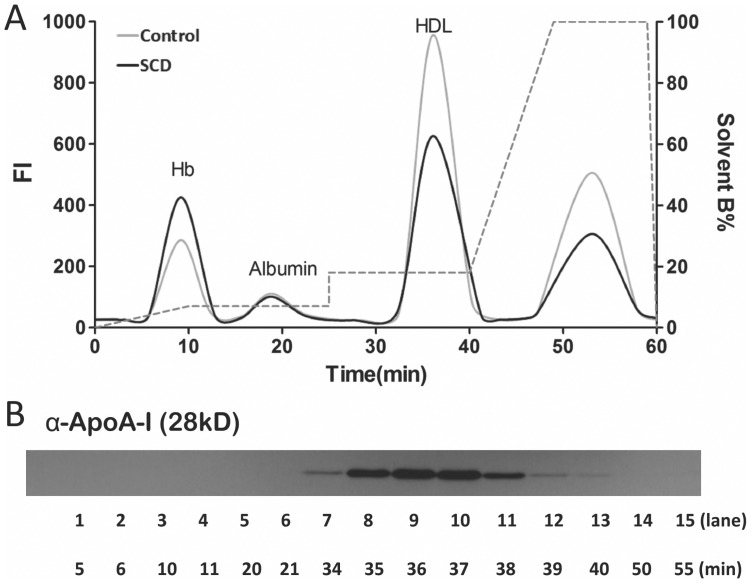
Effects of post-column reactor (PCR) on AE-HPLC separation of Hb, albumin and HDL. (A) DiI chromatogram showing that HDL can be separated from Hb, albumin and other lipoproteins albeit with broadened peaks. (B) Anti-apoA-I immunoblot of pooled fractions showing that apoA-I (i.e., HDL) eluted between 34 and 39 minutes in the modified protocol.

After establishing our new standard AE-HPLC protocol for isolating HDL we installed the PCR II and analyzed DiI-treated plasma to determine if the PCR II altered the chromatogram. **Figure 8A** shows that the PCRII with both pumps running broadens the peaks. Regardless, the immunoblot for apoA-I (**Figure 8B**) shows that HDL eluted at essentially same time frame as before (Figure 7).

**Figure 9:**
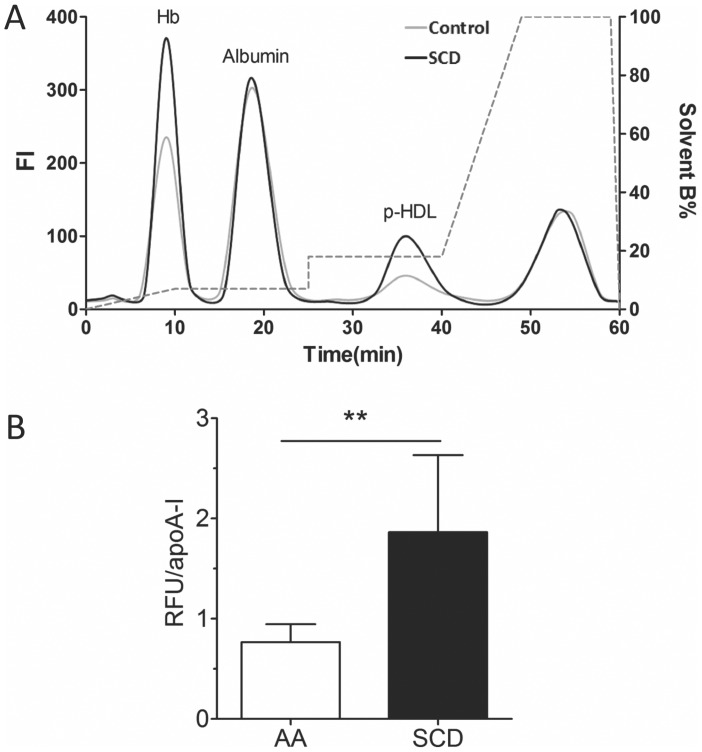
AE-HPLC-PCR Quantification of p-HDL in Control (AA) and SCD Plasma. (A) DCF chromatogram showing relative levels of DCF fluorescence (index of oxidizability) for p-HDL in control and SCD plasma. At 30–44 minutes, DCF fluorescence intensity in separated HDL in SCD plasma (black line) is greater than in control plasma (gray line). (B) Dividing DCF fluorescence intensity under these peaks by the subject’s plasma apoA-I concentration (µg/mL) yields relative fluorescence units (RFU) per apoA-I (µg/mL). P-HDL are increased in SCD subjects compared to control subjects (** = p<0.01, n = 6).

When the eluent from control and SCD plasma reacted first with Cu^2+^ and then Cu^2+^+DCFH marked increases in DCF fluorescence were observed in the HDL peaks in the SCD samples (**Figure 9A**). Dividing the fluorescence intensity in the area-under-the-peak by plasma apoA-I concentrations shows that SCD increased p-HDL levels when determined by the AE-HPLC-PCR method (**Figure 9B**).

**Figure 10:**
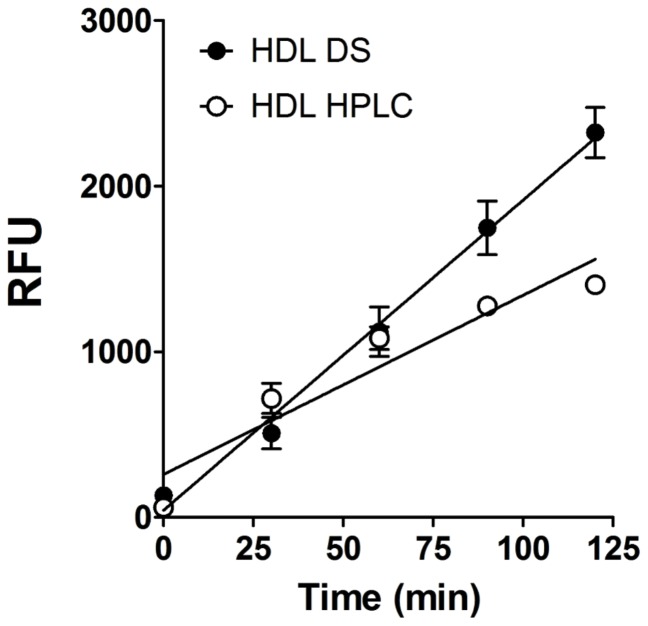
Effects of different methods for isolating HDL on rates of HDL oxidation determined by the plate assay. HDL was isolated from plasma by dextran sulfate-MgCl_2_ precipitation of apo B lipoproteins (black-filled circles, DS) or by AE-HPLC (white open circles). Rates of DCF oxidation in HDL isolated by DS and HPLC were determined by the plate assay (equal quantities of HDL cholesterol). Data were presented as mean ± SD of assays performed in triplicate. (y = ax+b; HDL DS: y = 18.728x+42.794, R^2^ = 0.9934; HDL HPLC: y = 10.817x+259.46 R^2^ = 0.9035).

**Figure 11:**
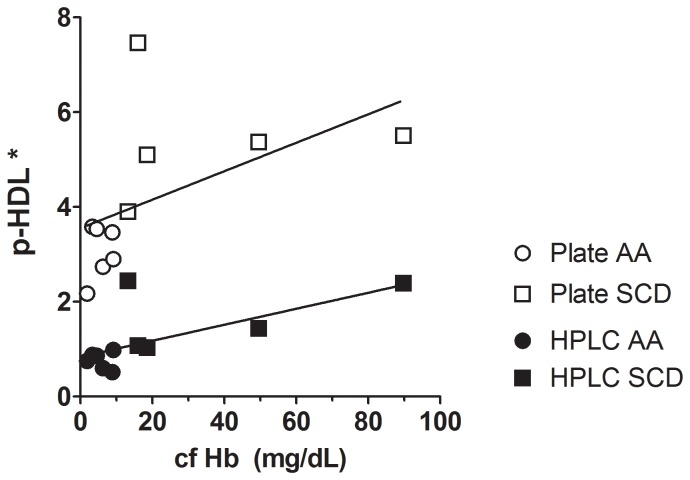
P-HDL plotted as a function of cf Hb: Plate vs. HPLC Assay. For plate assay, control AA p-HDL data (* RFU/µg HDL_c_⋅min) were plotted as open circles and SCD p-HDL data were plotted as open squares. For HPLC assay, controls (AA, meaning homozygous for hemoglobin AA) p-HDL data (* RFU/apoA-I (µg/mL)) were plotted as closed circles and SCD p-HDL data were plotted as closed squares. (y = ax+b; Plate assay: y = 0.03004x+3.554; HPLC assay: y = 0.01688x+0.84140). These data showed that p-HDL isolated by apo B precipitation was more sensitive to the effects of cf Hb and was more variable than p-HDL determined by the AE-HPLC-PCR assay.

To better appreciate the differences between the plate and AE-HPLC-PCR methods we compared the p-HDL HPLC data to the p-HDL plate data determined after DS precipitation (**Figure 10**). The curve for HDL isolated by DS precipitation had a higher slope than the curve for HDL isolated by HPLC method. Plots of p-HDL vs. plasma cf Hb determined by the plate assay and the AE-HPLC-PCR assay (**Figure 11**), revealed that p-HDL data generated by the plate assay appear to have greater scatter and more sensitive to cf Hb than p-HDL data generated by the HPLC.

## Discussion

The objective of this study was to develop an automated assay for quantifying p-HDL levels in plasma samples from SCD patients who are in chronic states of inflammation and oxidative stress. As SCD results in the release of pro-oxidative factors that have the potential to artificially increase p-HDL levels, it seemed reasonable to develop a new method for quantifying p-HDL free from the pro-oxidant effects of cf Hb, XO/X and albumin which is typically considered an antioxidant but when it binds lipid hydroperoxides or heme it can actually increase lipid peroxidation. Our data show that the AE-HPLC-PCR assay separates HDL from these plasma components making it less likely for them to influence HDL oxidation. Normalizing p-HDL AE-HPLC-PCR data by plasma apoA-I also seems to reduce variation compared to not normalizing (data not shown). The AE-HPLC-PCR assay yields lower p-HDL values than the modified plate assay and appears to be influenced less by cf Hb and plasma supernatants than our standard plate assay. Thus, the AE HPLC protocol yields lower p-HDL values that we think better reflect the impact of SCD on the proinflammatory character of HDL. Our studies show that the AE-HPLC protocol is essentially free of the influence of pro-oxidant factors commonly found in SCD supernatants. Accordingly, p-HDL levels measured by the AE-HPLC protocol may better represent circulating p-HDL levels in this unique patient population than p-HDL levels measured by the plate assay.

For years it has been recognized that HDL cholesterol was inversely related to the risk of atherosclerosis. However, re-analysis of data from the Framingham Heart studies revealed that nearly 40% of the clinically significant events, actually occurred in subjects who had normal HDL cholesterol levels. [31] Furthermore, analysis of the Air Force/Texas Coronary Prevention Study showed that event rates in individuals with normal HDL levels, who also received placebo, were nearly two-thirds the event rates in individuals with the lowest HDL levels (<34 mg/dL), the group most at risk. [12], [32] These clinical studies provided strong support for the idea that HDL protects cardiovascular health by mechanisms that do not appear to correlate with HDL_c_, the main biomarker currently used to monitor risk in patients. Fogelman and associates, prompted by studies showing that heart attacks occurred more frequently in the winter and during the flu season, [33] designed studies to determine why influenza infection increased cardiovascular events. They found that elderly patients with the flu were more likely to suffer acute myocardial infarction than age-matched controls who did not have the flu. [13], [34] They also observed that having the flu converted HDL into a proinflammatory lipoprotein that lost its atheroprotective properties and allowed more monocytes to enter the vessel wall and turn into foam cells than functional HDL [35], [36] and designed the first cell-free assay for quantifying p-HDL. [14] Part of the controversy concerning p-HDL’s role in vascular disease is whether it is permissive or causal. For example, does loss of HDL functionality permit oxidized LDL mechanisms to proceed unabated? Or does p-HDL directly cause atherosclerosis because it carries oxidized lipids or proinflammatory proteins. [37] Answers to this controversy can only come from designing and developing better and more specific assays of HDL functionality.

The cell-free p-HDL assay is a convenient assay with which to test HDL’s anti-inflammatory properties. While the original assay can be modified in different ways to test HDL anti-inflammatory properties, the basic assay calls for lipid-derived oxidants to be added to solutions of LDL or 1-palmitoyl-2-arachidonyl-3-phosphorylcholine as the oxidizable targets with which to test HDL’s ability to inhibit oxidation. As HDL loses functionality when oxidized, the cell-fee assay is considered an indirect measure of HDL oxidizability. In other words, what the cell-free assay shows is that p-HDL is easier to oxidize than anti-inflammatory HDL. We reasoned earlier that if HDL oxidizability was a measure of p-HDL, then HDL’s proinflammatory character could be tested with Cu^2+^, [10] which was easier to use and has a longer shelf-life than lipid-derived oxidants.

As we gained experience with the plate assay for quantifying p-HDL in different animal and human populations, we noted the assay yielded data that was, for the most part, quite consistent. However, whenever we analyzed SCD samples, not only were the p-HDL levels high but some of samples had very high values that could not be explained by chronic states of oxidative stress alone. Considering the possibility that cf Hb, which is often increased in SCD, might enhance oxidation of lipoproteins, we measured cf Hb and discovered that the concentration of cf Hb in SCD samples was increased and could directly influence p-HDL levels. Plasma free Hb contributes to p-HDL values in two ways. First, it accelerates HDL oxidation during the assay. Second, Hb and XO represent sources of oxidative stress *in vivo* that can increase oxidative modification not only of HDL but also other lipoproteins in the circulation. Any time a SCD patient experiences a vaso-occlusion crisis and/or tissue injury, these two components could team up to increase HDL oxidation *in vivo* as well as *ex vivo* during analytical assays.

To overcome such problems we needed to find a convenient way to purify HDL. Size exclusion (SE) and AE chromatography have both been used to separate lipoproteins. SE methods separate lipoproteins into individual peaks that are broad. Lipoproteins are released from the column based on size, where the largest, CM elutes first and smallest, HDL elutes last. In contrast, AE methods separate lipoproteins into their individual classes based on ionic charge resulting in lipoproteins eluting in sharper peaks than the peaks generated by SE chromatography. As the Hirowatari et al. [20] protocol separated lipoproteins quickly and in sharp peaks we chose to use their protocols. To make it easier to visualize eluting lipoproteins we used lipid soluble fluorescent dyes. This decision was based more on economics than on scientific reasons. Two lipid soluble dyes were investigated; Nile Red and, DiI. Nile Red is relatively non-fluorescent in simple aqueous buffers. It becomes fluorescent when incorporated in lipids. DiI is also a lipophilic fluorescent dye that has been known to readily incorporate into plasma membrane lipids and lipoproteins. Unpublished studies from our lab show that fluorescent intensity of both of these dyes in the lipoprotein fractions appear to track more with triglyceride than cholesterol. Although the dyes seemed to prefer triglyceride more than cholesterol, they were still convenient tools for monitoring separation. Extending interpretation of DiI and DCF lipoprotein data beyond monitoring profiles and/or quantifying HDL oxidizability would require additional studies that are designed to answer key analytical questions and focused experiments. Having said that, it should be noted that similar approaches have been used in capillary electrophoresis protocols, where sudan black B, [38] a well-known lipophilic dye was mixed with plasma samples to increase lipoproteins absorbance. Our decision to use DiI was based on availability and expense. While we fully anticipated that the lipoproteins would take-up DiI and DCF and fluoresce, we did not anticipate that albumin would bind DiI and DCF and elute close to HDL. These additional non-lipoprotein peaks in our chromatograms meant that the elution protocol had to be modified to improve HDL purification. This was accomplished by systematically increasing % B buffer in the early phases of the elution profile and using immunoblot analysis to confirm separation.

Bias between the two assays was estimated by plotting p-HDL vs cf Hb. Correlation studies revealed that the slope for the plate assay was greater than for the AE-HPLC-PCR assay. This is consistent with the idea that the plate assay yields higher p-HDL levels and is more influenced by cf Hb than the AE-HPLC-PCR assay. While p-HDL data from both assays resulted in a positive linear correlation with cf Hb concentrations, data scatter for the plate assay was greater than for the AE-HPLC-PCR assay. In future studies, measurements of p-HDL using both methods will be performed on multiple populations with larger sample sizes to determine if and the extent to which the matrix of supernatants in other patient populations (cardiovascular, diabetes, hypertension, autoimmunity) contribute to artificially increased p-HDL as we observed here for SCD.

It is possible that methodological differences between the assays may contribute to bias. While both assays use Cu^2+^ to oxidize HDL the plate assay measured HDL oxidation over 2 hr at room temperature. In contrast, the AE-HPLC-PCR assay measured HDL oxidation over a very short time; ∼3 min with Cu^2+^/3 min Cu^2+^+DCFH and at a higher temperature (50°C). The AE-HPLC-PCR assay purifies HDL prior to oxidation and fluorescent detection. Logically assays using purified HDL should yield data that more accurately reflects the oxidation status of HDL than the oxidation of HDL in the presence of a complex supernatant matrix as occurs in the plate assay.

Finally, while efforts here focused on factors that artificially increased p-HDL, no studies were performed to identify factors that could artificially decrease p-HDL. For example, when HDL from apoA-I deficient mice was measured for p-HDL levels, larger aliquots of the supernatant matrix were required to achieve a volume that contained 1 µg of HDL_c_ because total HDL_c_ was reduced in these mice. The larger sample volume naturally resulted in greater quantities of plasma proteins being added to the well of the plate assay (i.e., albumin, gamma globulins), which reduced p-HDL levels in the ApoA-I deficient mice below the p-HDL levels in healthy controls, which is inconsistent with the idea that HDL lacking apoA-I is proinflammatory (data not shown).

In summary, although the AE-HPLC-PCR assay for quantifying p-HDL requires specialized equipment, it is a simple assay that measures p-HDL by first purifying HDL from plasma samples, oxidizing it and then mixing it with DCF for quantifying oxidation. The AE-HPLC-PRC assay yields lower p-HDL values than the plate assay because it eliminates the effects of the supernatant matrix. This eliminates potential artifacts resulting from high plasma levels of cf Hb and XO. Additional animal and human population studies are necessary to determine the clinical usefulness of quantifying p-HDL by the AE-HPLC-PCR assay. On the basis that AE-HPLC-PCR quantifies p-HDL levels using purified HDL, it should yield data that more accurately reflect HDL’s proinflammatory state than assays using HDL in the supernatants of plasma samples after apo B precipitation. Logically, such extra steps should allow us to distinguish subtle differences in patient populations and make it easier to identify new mechanisms of atherosclerosis in a variety of clinical settings.

## Supporting Information

Figure S1 **Effects of systematic changes in B Buffer (6, 7 and 8%) on the elution of albumin and apoA-I.** (A) Immunoblots demonstrate that holding B Buffer at 7% provides for greater separation of albumin from apoA-I than the other % B Buffer protocols. (B) Chromatogram of UV absorbance of the separation of albumin from HDL. (C) Chromatogram of DiI fluorescence of the separation of albumin from HDL. (D) Changes in % B Buffer used to generate chromatograms B and C.
(TIF)

Figure S2 **Hb binds DiI. Increasing % B Buffer from 0–7% separates Hb from HDL.** (A) This UV chromatogram shows the absorbance (A_230 nm_) profile of a single peak that eluted 5 min after injection of Hb solution (30 µl, 100 mg/dL) mixed with DiI (10 µM). (B) This DiI chromatogram shows a single DiI fluorescent peak that elutes before 5 min as it did in chromatogram A.
(TIF)

Figure S3 **AE-HPLC analysis of HDL isolated by dextran sulfate-MgCl_2_ (DS) precipitation.** (A) This chromatogram shows that although apoB precipitation removed apoB containing lipoproteins it does not separate HDL from Hb and albumin. HDL in plasma supernatants after precipitation of apo B containing lipoproteins was treated with DiI (10 µM, final concentration) and then injected (30 µL) into the AE-HPLC. (B) Immunoblots show HDL isolated by DS/MgCl_2_ precipitation of apo B lipoproteins still contains Hb and XO.
(TIF)
