# Supplementary figures and images for: Anion Exchange HPLC Isolation of High-Density Lipoprotein (HDL) and On-Line Estimation of Proinflammatory HDL
Source: PLoS One. 2014 Mar 7;9(3):e91089. doi: 10.1371/journal.pone.0091089 (PMC3946658; doi:10.1371/journal.pone.0091089)

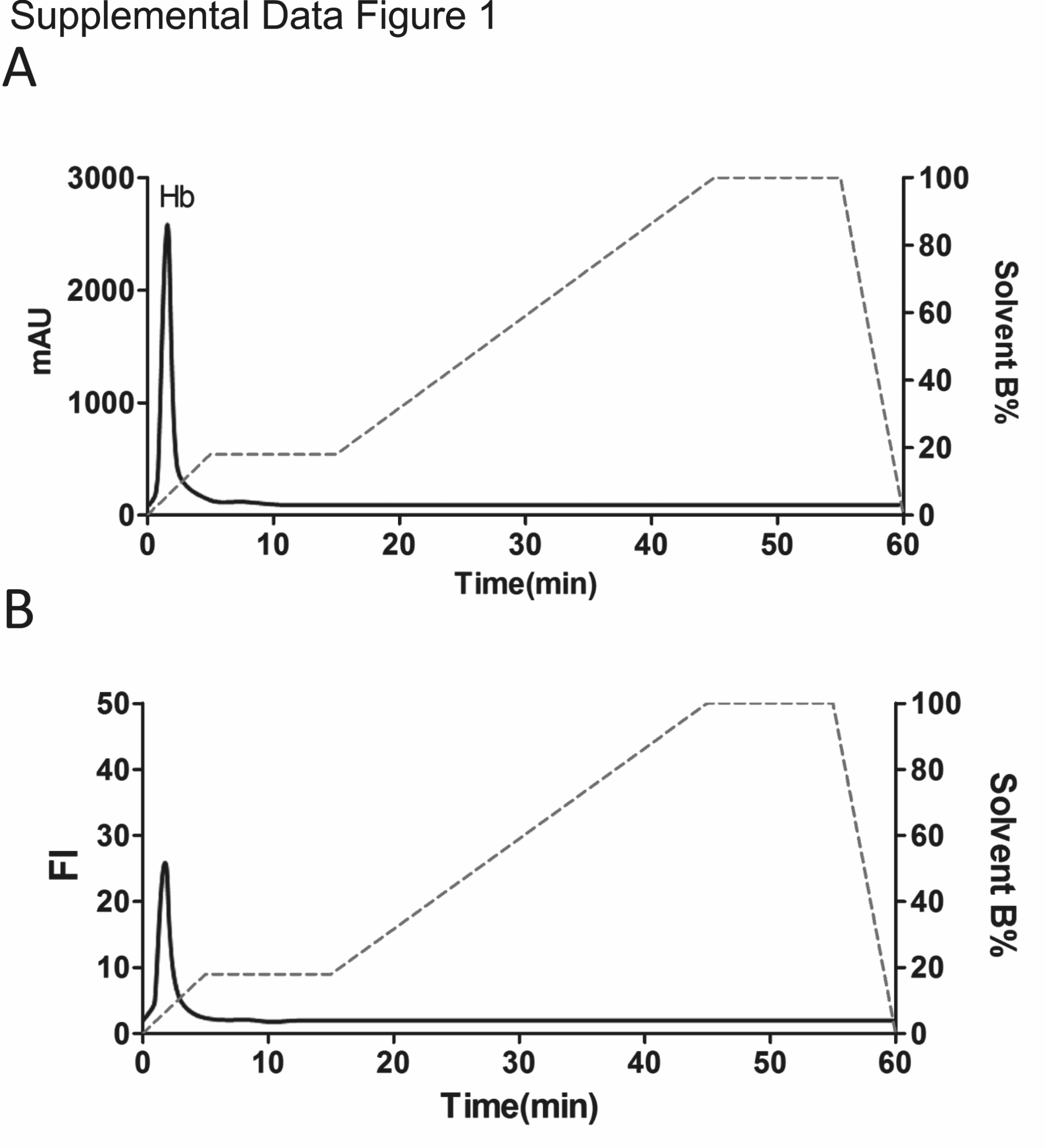

Supplement: Figure S1 — Effects of systematic changes in B Buffer (6, 7 and 8%) on the elution of albumin and apoA-I. (A) Immunoblots demonstrate that holding B Buffer at 7% provides for greater separation of albumin from apoA-I than the other % B Buffer protocols. (B) Chromatogram of UV absorbance of the separation of albumin from HDL. (C) Chromatogram of DiI fluorescence of the separation of albumin from HDL. (D) Changes in % B Buffer used to generate chromatograms B and C. (TIF) [file pone.0091089.s001.tif]

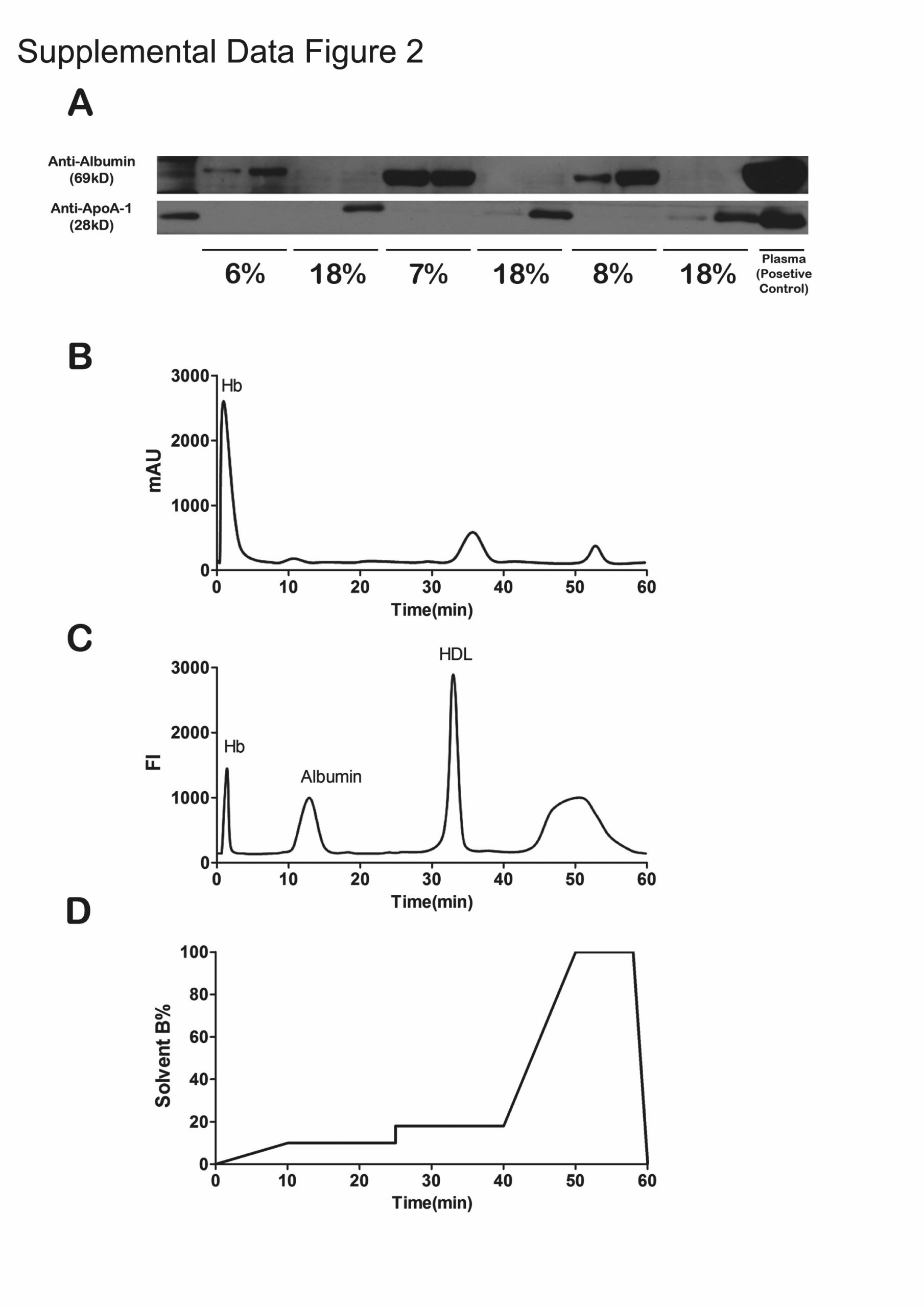

Supplement: Figure S2 — Hb binds DiI. Increasing % B Buffer from 0–7% separates Hb from HDL. (A) This UV chromatogram shows the absorbance (A230 nm) profile of a single peak that eluted 5 min after injection of Hb solution (30 µl, 100 mg/dL) mixed with DiI (10 µM). (B) This DiI chromatogram shows a single DiI fluorescent peak that elutes before 5 min as it did in chromatogram A. (TIF) [file pone.0091089.s002.tif]

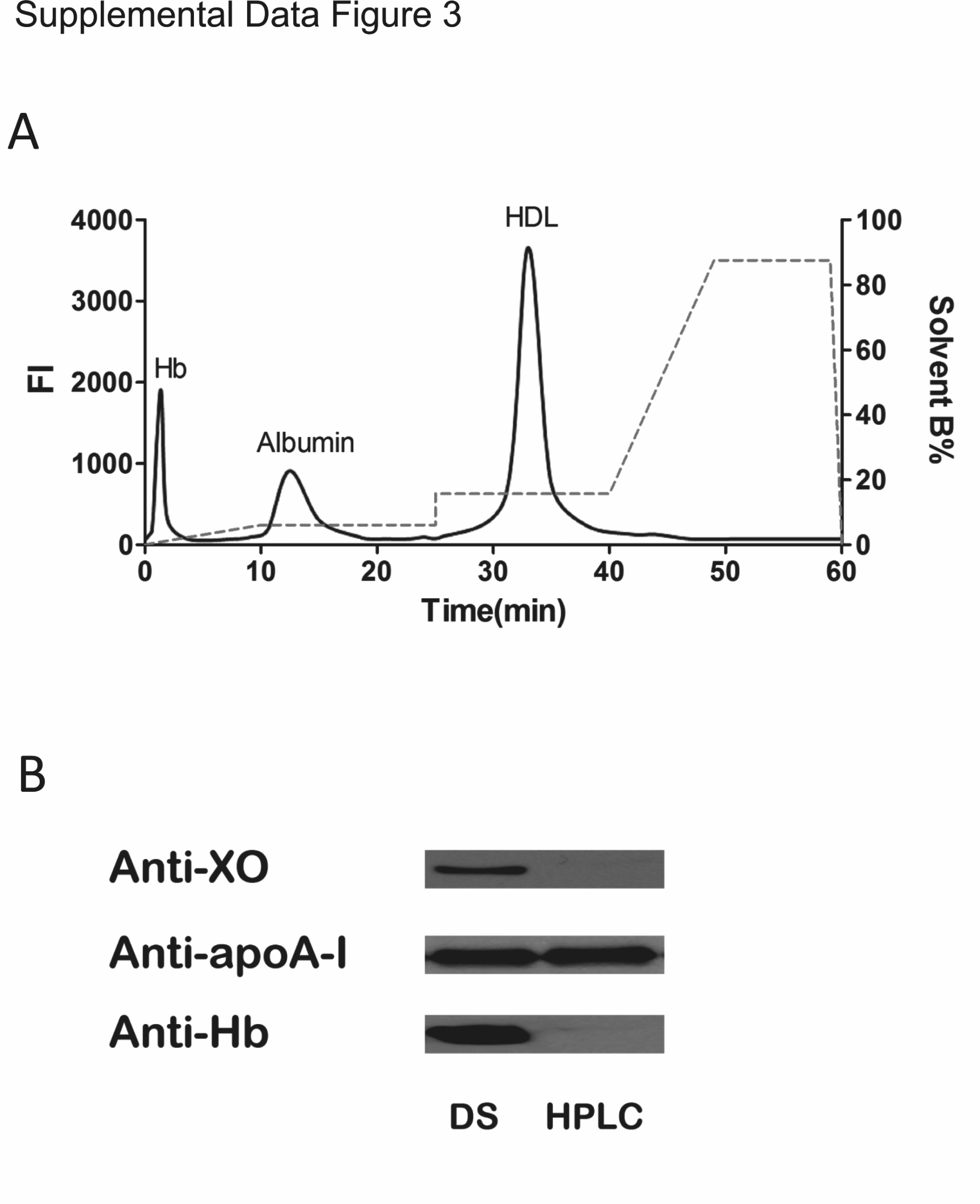

Supplement: Figure S3 — AE-HPLC analysis of HDL isolated by dextran sulfate-MgCl2 (DS) precipitation. (A) This chromatogram shows that although apoB precipitation removed apoB containing lipoproteins it does not separate HDL from Hb and albumin. HDL in plasma supernatants after precipitation of apo B containing lipoproteins was treated with DiI (10 µM, final concentration) and then injected (30 µL) into the AE-HPLC. (B) Immunoblots show HDL isolated by DS/MgCl2 precipitation of apo B lipoproteins still contains Hb and XO. (TIF) [file pone.0091089.s003.tif]
